# "Before" and "after": Investigating the relationship between temporal connectives and chronological ordering using event-related potentials

**DOI:** 10.1371/journal.pone.0175199

**Published:** 2017-04-03

**Authors:** Stephen Politzer-Ahles, Ming Xiang, Diogo Almeida

**Affiliations:** 1 Faculty of Linguistics, Philology & Phonetics, University of Oxford, Oxford, United Kingdom; 2 NYUAD Institute, New York University Abu Dhabi, Abu Dhabi, United Arab Emirates; 3 Linguistics Department, Chicago, University of Chicago, United States of America; 4 Psychology Program, New York University Abu Dhabi, Abu Dhabi, United Arab Emirates; University of Nottingham, UNITED KINGDOM

## Abstract

Sentence-initial temporal clauses headed by *before*, as in "Before the scientist submitted the paper, the journal changed its policy", have been shown to elicit sustained negative-going brain potentials compared to maximally similar clauses headed by *after*, as in "After the scientist submitted the paper, the journal changed its policy". Such effects may be due to either one of two potential causes: *before* clauses may be more difficult than *after* clauses because they cause the two events in the sentence to be mentioned in an order opposite the order in which they actually occurred, or they may be more difficult because they are ambiguous with regard to whether the event described in the clause actually happened. The present study examined the effect of *before* and *after* clauses on sentence processing in both sentence-initial contexts, like those above, and in sentence-final contexts ("The journal changed its policy before/after the scientist submitted the paper"), where an order-of-mention account of the sustained negativity predicts a negativity for *after* relative to *before*. There was indeed such a reversal, with *before* eliciting more negative brain potentials than *after* in sentence-initial clauses but more positive in sentence-final clauses. The results suggest that the sustained negativity indexes processing costs related to comprehending events that were mentioned out of order.

## Introduction

One of the hallmarks of human language is the ability to talk about events that are displaced in time and/or space from the speaker; this includes past events, events that have not happened yet, and possible events that did not actually happen [1]. Temporal connectives like *before* and *after* pose a special challenge to the language comprehension system, as they express relationships between multiple events. Since events may have complicated relationships—for instance, one event may begin after but end before another—which in turn affects the way temporal expressions are used [2–6], the comprehension of temporal expressions, therefore, requires sophisticated temporal alignment between multiple events.

A well-known phenomenon in the comprehension of temporal connectives is that English sentences beginning with a temporal clause headed by *before* (1a) engender greater processing cost than those beginning with a temporal clause headed by *after* (1b).

(1) a. Before the scientist submitted the paper, the journal changed its policy.
  b. After the scientist submitted the paper, the journal changed its policy.

In the seminal study on this phenomenon using event-related brain potentials (ERPs), which provide a measure of neural activity recorded at the scalp with precise temporal accuracy, [7] showed that *before* sentences like (1a), relative to *after* sentences like (1b), elicited a negative-going ERP component over anterior sites on the scalp, which was sustained over the whole sentence. Anterior negativities are often argued to be elicited by stimuli or cognitive tasks which require greater working memory resources ([8–10], among others). The authors propose that the increased negativity elicited by *before* sentences is related to working memory demands and additional computation associated with having to construct a conceptual model in which the events occur in a different order than the one in which they were presented in the sentence. In other words, (1a) describes a situation in which the first event that happened is the journal's changing its policy, and the second event is the scientist's submitting her paper; in the sentence, however, these two events are mentioned in the opposite order (counter-chronological order of mention), which leads to more difficult processing.

A variety of other research paradigms have shown similar costs for *before* sentences relative to *after* sentences. In behavioral experiments, sentences in which the order of mention of two events is different from the conceptual order in which they actually occurred are recalled less accurately [11], are read more slowly [12], and are re-enacted less accurately by children in some experiments [13, 14] (see, however, [15, 16]). Using ERPs, [17] finds that an N400 effect related to a truth-value manipulation was attenuated in *before* sentences compared to *after* sentences, suggesting that real-world event knowledge was recruited in a different way in the context of *before* compared to *after*. With functional magnetic resonance imaging (fMRI), [18, 19] showed greater hemodynamic activation in the caudate nucleus and left middle frontal gyrus (which, together, may be involved in maintaining and manipulating representations in working memory) for *before* sentences compared to *after* sentences in healthy adults.

The processing cost for *before* clauses in this line of research has been generally attributed to the non-isomorphic mapping between the order of mention in the linguistic string and the ordering of the actual events in the real world. However, another possibility is that the processing difference between *before* and *after* is rooted in the semantic and pragmatic differences between the temporal expressions *before* and *after* themselves. There are several asymmetries between the semantics of *before* and of *after* [3–5, 20], but the most important for present purposes is the difference in *veridicality*: *after* entails that the temporal clause event happened, and *before* does not. That is to say, the *after* clause in (1b) necessarily means that the scientist did ultimately submit her paper (it entails that the event described in the temporal clause is veridical). On the other hand, the *before* clause in (1a) is ambiguous: it might be the case that the scientist submitted her paper, but it might not, as in (2).

2 Before the scientist submitted the paper, she ripped it up and threw it away.

Thus, as a result of the different entailment pattern of *before* as opposed to *after*, a *before* clause introduces temporary ambiguity as to whether or not the event described actually happened. This point was also noted by [21] and [22], who propose that the sustained ERP negativity observed by [7] may be due not to the difficulty of realizing the conceptual order when it mismatches the order of mention, but rather may be due to ambiguity of the *before* clause and the concomitant working memory costs associated with holding multiple possible readings in working memory until it is possible to decide whether or not the event described in the *before* clause actually occurred. Consistent with this account, [21] replicated the sustained negative effect with sentences like (1a,b), but also showed that the effect disappeared when participants instead read sentences like (3a,b) in which real-world knowledge makes it clear that the event actually happened.

3 a. Before the Second World War broke out, John worked at a small factory.
  b. After the Second World War broke out, John worked at a small factory.

While this finding provides suggestive evidence that the sustained negativity may have been due to ambiguity, some details of the results are surprising. Notably, the sustained negativity in ambiguous sentences—and the corresponding *lack* of sustained negativity in unambiguous sentences—emerged right at the beginning of the sentences; there was no point early in the epoch where unambiguous *before* clauses elicited a transient negativity. The point at which the unambiguous clauses would have been disambiguated to a veridical reading, however, was generally later in the clause, presumably around the temporal clause verb (for example, until the verb "broke out" was read, (3b) could have had an anti-veridical continuation such as "Before the Second World War *caused the extinction of humankind*, *a peace treaty fortunately was signed*"). The fact that the unambiguous temporal clauses showed no sustained negativity at all, rather than an early emergence and later disappearance of a negativity, suggests that the lack of effect for these clauses may have been due to strategic factors as well as to unambiguity.

At present, therefore, it is difficult to adjudicate between the account of the sustained negativity based on counter-chronological order of mention and that based on the ambiguity of the event described by *before*, as both accounts make the same predictions for sentences like (1a,b) without real-world disambiguating information. However, these accounts can be straightforwardly tested by examining sentences in which the temporal clause follows rather than precedes the main clause, such as (4a,b), which describe the same situations as (1a,b) but in the opposite order of mention:

4 a. The journal changed its policy after the scientist submitted the paper.
  b. The journal changed its policy before the scientist submitted the paper.

In this case it is the order of mention in the *after* sentence, not the *before* sentence, that is counter-chronological. Thus, under the hypothesis that the sustained negativity is based on the incongruence between the conceptual order of the events and their order of mention, these sentences should show the opposite of the effects described above: over the temporal clause (*before/after the scientist submitted the paper*), an increased negative ERP should be observed for *after* clauses compared to *before* clauses (this prediction is also made by [23], p. 28). On the other hand, the hypothesis that the sustained negativity is based on the ambiguity of the *before* clause does not predict such a reversal of the ERP effect. Rather, under such an account, one would make the following predictions. First, it is possible that *before* clauses would still elicit a greater negativity than the *after* clauses. This is because changing the order of mention, as in (4b), does not necessarily eliminate the veridicality ambiguity in the before-clause. For instance, (4b) is still ambiguous as to whether the scientist actually submitted his paper or not (maybe the journal changed its policy and then the scientist grumbled about the hassle but submitted her paper anyway, or maybe the journal changed its policy and then the scientist decided not to submit after all). It is also possible, however, that seeing the main clause first helps to reduce the ambiguity (if not completely eliminating it), since a comprehender would have more information to work with when incrementally making veridicality inferences about the before-clauses. In this case the *before* and *after* clauses should pattern similarly to each other. Crucially, in neither case would a larger negativity on the *after* clauses relative to the *before* clauses be predicted. Thus, while sentence-final temporal clauses like those in (4a-b) do not necessarily test a positive prediction of the veridicality-based account (since that account does not necessarily predict *before* clauses to be more difficult than *after* clauses in sentence-final temporal clauses), they do at least test a positive prediction of the order-of-mention account that is not made by the veridicality-based account.

Thus far, only behavioral experiments have examined sentence-final temporal clauses like (4a,b). Most such studies have found a reversal (in terms of reading times [12], act-out accuracy in children [14], or recall accuracy [11]) as predicted by counter-chronological order of mention account: better performance in sentence-final *before* clauses compared to sentence-final *after* clauses. On the other hand, adults in the study by [14] showed better performance on *after* than *before* across the board, regardless of the order of mention, consistent with *before*-ambiguity account. Thus, the extant behavioral literature is somewhat equivocal between the two accounts. The present study tests the order-of-mention and ambiguity hypotheses by examining ERPs elicited while participants read sentences with sentence-initial temporal clauses like (1a,b) and sentence-final temporal clauses (4a,b) for comprehension.

## Methods

### Participants

Twenty native speakers of English (14 women, mean age = 26, SD = 8.2, range 18–47) were included in the final analysis. All were right-handed as assessed by the Edinburgh Handedness Questionnaire [24]. All participants provided their informed consent and were paid for their participation, and experimental procedures were approved by the Institutional Review Board of New York University Abu Dhabi. Detailed demographic information about the participants is available in S1 Spreadsheet. An additional nineteen participants took part in the study but were not included in the final data analysis: ten were removed because of excessive artifact in their data (<12 trials in one or more conditions), six for being early bilinguals, one for being left-handed, and two to ensure that the same number of participants completed each list of the design (see section 2.3, Procedure).

While the proportion of participants removed from data analysis for artifact was high compared to many studies, this is not surprising given that we analyzed a large epoch (see section 2.4, Data acquisition and analysis) and had to exclude trials of data including artifacts anywhere within the relatively long epoch. As for the decision to exclude early bilinguals, given the very heterogeneous language profile of our participant population in Abu Dhabi, at the outset of the study we recruited anyone who self-reported as a native English speaker because we were worried we would not find sufficient participants if we only used early monolinguals (who, according to self-report, were not exposed to a second language until adulthood). When it became clear later in the data collection process that there would be enough early monolingual participants, we decided to exclude bilinguals from the analysis given that the different temporal clause structures in their language (for instance, many of these participants were speakers of languages with head-final temporal clauses, where the equivalent of *before* or *after* would come at the end of the clause) may influence their processing strategy. Nonetheless, exploratory analysis of the dataset with these participants included showed the same pattern of results as that reported below. As for participants removed to balance the number of participants per list, this was done by removing the participants with the lowest number of trials left from the lists that had extra participants. Exploratory analysis of the dataset with these two participants included showed the same pattern of results as that reported below. To examine the pattern of results with more participants included, we also conducted an exploratory re-analysis of the data in which we first artifact-corrected the data using independent components analysis, which allowed us to retain more participants and trials in the dataset; this analysis yielded a qualitatively similar pattern as that described below, and is reported in more detail in S2 Text.

### Materials

The experimental stimuli comprised 154 two-clause sentences of the format shown in (1) and (4). The materials were adapted from [19] and [21] (by taking the temporal and matrix clauses from these items and, for sentence-final temporal clause conditions, reversing their order). Each item comprised two clauses which were not causally related and did not contain any pronoun-antecedent dependencies across clauses (of the original 160 items, six which were later noticed to include dependencies were excluded from data analysis). The four conditions were created by heading the temporal clause with either *before* or *after*, and by placing the temporal clause either before the main clause (and following it with a comma) or after the main clause. Thus the experiment followed a 2×2 design: Connective (*before* vs. *after*) × Structure (sentence-initial temporal clause vs. sentence-final temporal clause). The items were organized into four lists in a Latin square design. The full list of critical stimuli, along with ratings of how likely each sentence was to be interpreted veridically (procedure described below) is available in S2 Spreadsheet.

An additional 160 sentences from a separate experiment, including different kinds of *wh*-islands and resumptive pronouns, served as fillers. These sentences (e.g. "What does the detective {think that/wonder whether} Paul took {Ø/it} from the store?") included both subjacency and resumptive pronoun errors (out of the design illustrated in the previous example sentence, there were 40 trials of each type, yielding 80 or 120 sentences with grammatical errors depending on whether "What does the detective wonder whether Paul took it from the store?" is judged grammatical or ungrammatical). Participants were informed at the beginning of the experiment that some sentences may "have something wrong with them", but were instructed to try their best to comprehend each sentence anyway.

### Procedure

Participants were seated in an electrically-shielded and sound-attenuated booth, in front of a 59 cm, 1920×1080 pixel LCD monitor. They read the 320 stimulus sentences (in yellow 32-point Courier New font on a black background) word-by-word for comprehension as the electroencephalogram (EEG) was recorded. The experiment was controlled using Presentation (Neurobehavioral Systems). Each trial began with a 64-point fixation cross presented at the center of the screen for 500–800 ms, after which the sentence was presented word by word (for the filler sentences, some short phrases were presented in single chunks). Each word remained on screen for 300 ms (except for the final word of the sentence-initial temporal clauses, which was presented for 500 ms together with a comma, and for the final word of each sentence, which was presented for 800 ms together with a period; these increased durations were used to accommodate for potential end-of-clause wrap-up processes) and followed by a blank screen for 200 ms.

Participants’ task during the experiment was to answer comprehension questions about the sentences. [23] (p. 28) suggests that the sustained negativity [7] observed for *before* sentences in which order-of-mention mismatches the temporal order of the events may actually be a spurious effect introduced by a comprehension task in which participants must explicitly represent the events in the correct temporal order. This concern is less likely to apply to the present design, however, as our comprehension questions did not directly probe the order of the events, and only one comprehension question (that for item 26; all stimuli are available in S2 Spreadsheet) asked about temporal information at all. (It is possible, however, that participants may still have expected upcoming comprehension questions to probe the order of events, and may have processed the sentences accordingly; we thank an anonymous reviewer for this raising this possibility.) We also note that [17] directly compared ERP responses in participants with and without explicit comprehension tasks and did not find evidence that the temporal connective effect differed as a function of task.

One-third of the items were followed by a comprehension question, which probed various portions of the sentence. For each question, two possible answers were displayed on the screen (the sides were determined randomly at runtime), and participants indicated the correct answer with their right hand using a gamepad. Trials with no comprehension question were simply followed by the message "(press any button to continue)". In either case, the next trial began as soon as the participant pressed a button.

The 320 items were presented in a fully random order after a three-sentence practice. The experiment was divided into five blocks, with 64 sentences per block, and optional break times in between.

Overall, the experimental session (including the completion of consent and demographic forms, applying the EEG cap, the EEG experiment, a working memory test [see below], and debriefing) took less than 1.5 hours per participant.

### Working memory test

Following previous ERP studies on temporal connective processing, we also tested whether the observed ERP effects correlate with individual differences in working memory. After the end of the EEG experimental session, participants completed a computer-mediated version of the reading span task described by [25] (see also [26]) to measure individual differences in working memory. The task was administered using Paradigm (Perception Research Systems, Inc.). Participants saw 12 item sets, each consisting of two to five trials. On each trial, the participant saw a visually-presented sentence followed by a "?" and a capital letter. Sentences were either conceptually anomalous (e.g., "During the week of final spaghetti, I felt like I was losing my mind") or conceptually acceptable (e.g., "During the winter you can get a room at the beach for a very low rate"). The participants' task was to read the sentence aloud and then make an acceptability judgment using the mouse. After making the judgment, the participant was to say the following letter aloud, after which the next trial was presented. After completing all two to five trials in an item set, the participant was asked to recall the final letters of each trial in that item set, in order. Item sets and trials were presented in the same order for all participants. Within an item set, no two trials had the same letter following the sentence. Before beginning the test, participants completed a practice block consisting of three two-trial item sets.

Each participant's performance on the recall portion of the span task was scored according to the partial-credit unit scoring procedure described by [26]. In this procedure, each item set gets a score reflecting what proportion of trials the participant recalled correctly in that item set (e.g., a participant correctly recalling 2 trials out of 5 would receive a score of .4 for that item set) and the scores of the 15 items are then averaged, yielding an aggregate score between 0 and 1 for each participant, with higher scores reflecting greater recall accuracy. Each participant's accuracy on the secondary processing task (acceptability judgments) was also calculated as the proportion of trials with correct performance. Data for one participant who achieved perfect recall but was placing his fingers on the keyboard to remember the letters, was replaced with the mean of other participants' scores. Finally, recall and processing scores were averaged to yield a composite score. Individual participants' recall and accuracy scores are shown in S1 Spreadsheet.

### Data acquisition and analysis

The EEG was continuously sampled (1000 Hz, 0.1–250 Hz analog filter) from 34 Ag/AgCl electrodes (actiCAP, Brain Products) in a 10/20 layout. FCz served as the online reference and AFz as the ground. Up to three bad channels per participant, if present, were interpolated offline, and the continuous data were then re-referenced to the average of both mastoids and segmented into epochs from -200 ms to +2500 ms relative to the onset of the temporal connective. This epoch window was chosen to encompass the shortest temporal clauses. Trials containing artifact were removed from subsequent analysis based on visual inspection. The artifact-free trials were baseline-corrected using a -200 to 0 ms pre-stimulus baseline and subjected to a 30 Hz low-pass filter (Hamming windowed-sinc FIR filter, 440 samples filter order, in EEGLAB [27]).

Statistical analysis was carried out using spatiotemporal clustering [28], implemented in the FieldTrip toolbox [29]. (For the sake of comparison with previous studies we also carried out a traditional analysis based on mean amplitudes. This analysis is reported in S1 Text.) Compared to traditional analysis of mean ERP amplitudes over pre-defined time windows and channel selections, this method is more neutral to researcher choices, and also addresses the multiple comparisons problem. Spatiotemporal clusters between -200 and +2500 ms with a significant Connective×Structure interaction were identified, using a cluster α level of 0.3 (based on our *a priori* expectation to observe effects that would be subtle in amplitude but long-lasting). Cluster-level p-values were estimated from 500 random permutations of the data. The Connective×Structure interaction was coded such that a negative test statistic would represent a cluster where the simple effect of Connective (*before–after*) was more negative in sentence-initial clauses than sentence-final clauses, and a positive test statistic would represent a cluster where the effect was more positive. (See http://www.fieldtriptoolbox.org/faq/how_can_i_test_an_interaction_effect_using_cluster-based_permutation_tests regarding the coding of factorial interactions in FieldTrip; for a similar analysis see [30]).

## Results

### Behavioral

The lowest accuracy score on the comprehension task for any participant was 83.3%, indicating that participants were attending to the stimuli. Mean accuracy was 93.2% for sentence-initial *after* items, 96.0% for sentence-initial *before*, 89.5% for sentence-final *after*, and 88.9% for sentence-final *before*. A generalized (logistic) linear mixed-effects model with fixed effects of Connective, Structure, and their interaction, and crossed random intercepts for participants, items, and lists [31] yielded a marginal Connective×Structure interaction in model comparison (χ^2^(1) = 0.093). The interaction indicated that accuracy was marginally higher for *before* than *after* sentences when the temporal clause was sentence-initial (*b* = 0.77, *z* = 1.78, *p* = .072) but not when the temporal clause was sentence-final (*b* = -0.09, *z* = -0.30, *p* = .768); or, alternatively, that accuracy was significantly higher for sentences with sentence-initial than sentence-final temporal clauses when the connective was *before* (*b* = 1.49, *z* = 3.69, *p* < .001) but only marginally so when the connective was *after* (*b* = 0.63, *z* = 1.90, *p* = .057).

### ERPs

After artifact exclusion, the minimum number of trials retained in any cell was 14 (see S1 Spreadsheet; 65% of sentence-initial *before* clause trials were kept, 65% of sentence-initial *after*, 80% of sentence-final *before*, and 82% of sentence-final *after*). A generalized linear mixed model showed that significantly more trials were retained in sentence-final temporal clause configurations than sentence-initial clause configurations (χ^2^ (1) = 22.62, *p* < .001), but there was no difference based on Connective and no interaction (*p*s > .615).

**Fig 1:**
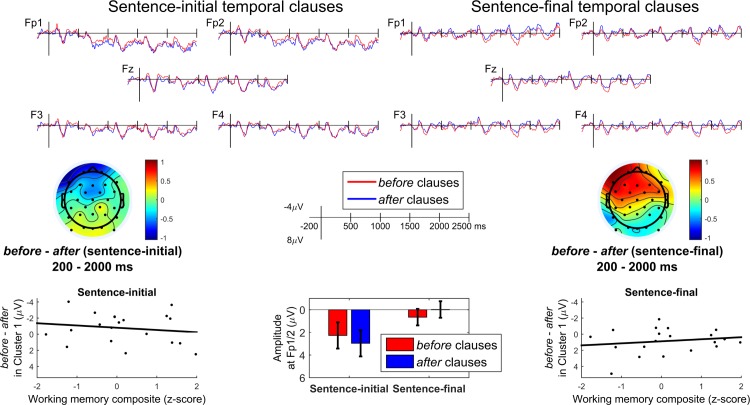
ERP results. ERPs at frontal electrodes (top portion) for the sentence-initial temporal clauses (left) and sentence-final temporal clauses (right). Topographic maps of the *before*–*after* difference averaged over the 200–2000 ms time window are shown below, as well as barplots of this difference at the front-most electrode region (with 95% Cousineau-Morey intervals [32]). The bottom left and right portion of the figure shows the correlation between working memory scores and ERP effect sizes for both the sentence-initial temporal clauses (left) and sentence-final temporal clauses (right).

The ERPs for each condition at a selection of frontal electrodes, along with topographic maps for the mean amplitude across most of the epoch, are shown in Fig 1; the ERP averages and raw data are available at https://osf.io/gevfz/. The figure suggests that in sentence-initial position, clauses with *before* elicited a subtle but sustained anterior negativity relative to clauses with *after*, whereas in sentence-final position, it is clauses with *after* that elicit a negativity relative to clauses with *before*. Statistical analysis confirmed these observations.

**Fig 2:**
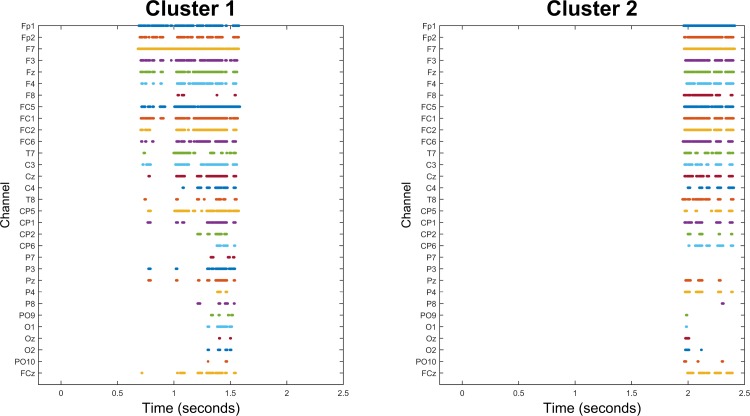
Cluster extents for the interaction effect. Raster plots showing the spatiotemporal extents of the most significant interaction cluster (*p* = .084, left side) and second-most significant interaction cluster (*p* = .133, right side). Each row represents a channel, and each colored dot along that row represents a timepoint during which that channel was included in the cluster.

The cluster analysis for the Connective×Structure interaction yielded a marginal negativity (*p* = .084) driven by a cluster with the spatiotemporal distribution illustrated in the raster plot [33] on the left side of Fig 2; i.e., it extended from about 700 to about 1600 ms in the frontal channels, was more sustained in the left channels than the right channels, and emerged in centro-posterior channels only towards the end of this time window. Averaging together the amplitudes of all <channel,time> samples within this cluster and conducting pairwise *t*-tests on the averages revealed that the ERPs elicited by sentence-initial *before* clauses were more negative than those elicited by sentence-initial *after* clauses (*t*(19) = -2.22, 95% CI = -1.76…-0.05, *p* = .039), whereas sentence-final *before* clauses were marginally more *positive* than sentence-final *after* clauses (*t*(19) = 1.96, 95% CI = -0.05…1.52, *p* = .065). Note that this is not a non-independent analysis [34], as the follow-up analysis tested simple effects, rather than the interaction test which was used as the basis for identifying the cluster. The purpose of the follow-up tests was not to reiterate the significance of the interaction, but to further clarify the nature of the interaction (e.g., while in our dataset the interaction emerged because the *before*-*after* effect was negative in sentence-initial clauses and positive in sentence-final clauses, it could have been the case that both effects were negative and the sentence-initial effect was simply more negative; it also could have been the case that neither simple effect was significantly different than zero even though they were different from one another).

While the crucial interaction did not reach statistical significance at the traditional .05 alpha level, we nonetheless take it to be consistent with the presence of opposite patterns of negativity for the sentence-initial and sentence-final temporal clauses. First of all, statistical significance is not intended to be treated as a bright line for determining whether an effect is real or not ("Scientific conclusions … should not be based only on whether a *p*-value passes a specific threshold" [35]). Secondly, the interaction was based on a specific *a priori* prediction and conceptually replicating a known effect; as such, it is in fact more trustworthy than a significant but unexpected effect.

There was another negative trend (*p* = .133) due to a cluster that emerged later, as illustrated in Fig 2. In this cluster, sentence-initial *before* clauses were marginally more negative than sentence-initial *after* clauses (*t*(19) = -1.77, 95% CI = -1.64…0.14, *p* = .092) and sentence-final *before* clauses significantly more positive than sentence-final *after* clauses (*t*(19) = 2.44, 95% CI = 0.12…1.57, *p* = .025).

There were no other noteworthy trends in either direction (*p*s > .262). There were also no significant main effects of Connective (*p*s > .487). There were significant main effects of Structure in both directions, but these are not of interest because they involve direct comparison across clauses at different portions of the sentence.

#### Correlation analysis with working memory

The relationship between individual working memory and the Connective effect (operationalized as the mean amplitude of the *before*–*after* difference within the interaction cluster, calculated separately for sentence-initial and sentence-final temporal clauses) is illustrated in Fig 1. While there was an apparent numerical trend towards opposite memory effects for sentence-initial versus sentence-final clauses, none of these were significant. Specifically, there was no significant correlation between working memory and effect size either for sentence-initial temporal clauses (*b* = 0.28, *R*^2^ = .02, *F*(1,18) = 0.46, *p* = .508) or for sentence-final temporal clauses (*b* = -0.25, *R*^2^ = .03, *F*(1,18) = 0.57, *p* = .461), nor did Structure and working memory significantly interact in a linear mixed model with random intercepts for participants (χ^2^(1) = 1.76, *p* = .185). Furthermore, the trends were in the opposite direction of those reported earlier: here, participants with higher working memory had effects nearer to zero, whereas for [7] and [21] participants with higher working memory had larger (more negative) effects.

#### The role of veridicality

While the main ERP analysis did not show evidence that the sustained negativity on sentence-initial *before* clauses was due to ambiguity in the veridicality of the clause, we hypothesized *post hoc* that such an ambiguity effect may be observable on an item-to-item level if we took into account variation in the veridicality bias of each item—for example, real-world knowledge (as in example (3)) or entailments (as in example (2)) may in some sentences disambiguate the veridicality or anti-veridicality of the event described in the temporal clause, and implicatures or plausibility-related priors may make the veridicality or anti-veridicality seem more likely even if the sentence is not fully unambiguous. To that end, we collected norming data to evaluate each item's likelihood of being interpreted as veridical, and regressed the item-wise ERP averages on these ratings to see whether more ambiguous items would yield more negative ERP effects.

Ratings were collected via Amazon Mechanical Turk, with each item being presented as a single Human Intelligence Task (HIT). In each HIT, participants were shown the sentence up to the end of the temporal clause (which means they were shown a sentence fragment in the case of sentence-initial temporal clauses, but shown a full sentence in the case of sentence-final temporal clauses) and asked to evaluate, on a scale from 1 (very unlikely) to 5 (very likely), the likelihood that the event described in the temporal clause indeed happened. Each HIT was completed by six unique workers, and a worker was allowed to complete multiple HITs, such that one worker may have contributed responses to multiple items, but may only have contributed one response to any given item. Items were normed in both their sentence-initial *before* versions and their sentence-final *before* versions, but not in either *after* version, since we did not predict variation in veridicality bias for *after* sentences. In order to exclude workers from contributing ratings to an item which they had already rated in the other condition, the HITs were divided into two Latin square lists which were presented on two subsequent weekdays, at about the same time of day (08:00–9:00 EST). Overall, 1920 ratings were collected (160 items × 2 conditions × 6 workers), 1412 of which were from monolingual English speakers (according to self-report); the others were removed from further analysis. For each item, the ratings from the self-reported monolingual English speakers were averaged to yield an average veridicality bias rating for that item.

**Fig 3:**
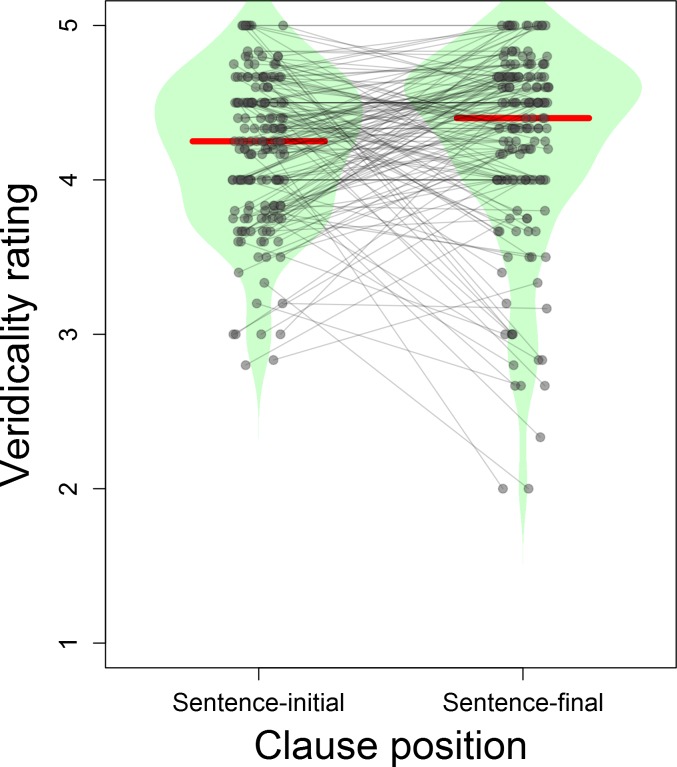
Veridicality ratings. Veridicality ratings for temporal clauses in sentence-initial and sentence-final positions. 1 indicates a response that the temporal clause event is "very unlikely" to have occurred, and 5 that the event was "very likely". Each point represents the mean veridicality rating for one item. The shaded violin-shaped regions represent smoothed kernel density of the veridicality ratings for each condition, and the horizontal red lines represent the median veridicality rating for each condition.

Violin plots of the item-wise average ratings are shown in Fig 3; the ratings for each item are given in S2 Spreadsheet. It is apparent that sentence-final temporal clauses elicited slightly higher veridicality ratings on average than sentence-initial ones (4.25 vs. 4.19), although this difference was not significant (*t*(159) = 0.94, 95% CI = -0.06…0.17, *p* = .347). Sentence-final temporal clauses also had a larger standard deviation of ratings (0.63 vs. 0.49). The wider range of ratings for sentence-final clauses is likely due to the availability of more context (the matrix clause as well as the temporal clause) which, rather than always disambiguating to a veridical reading, may sometimes have biased participants to make anti-veridical readings. (Note that, while none of our items explicitly disambiguated to anti-veridical readings—i.e., there were no items like "The police defused the bomb before it exploded"—other aspects of the full sentence may nonetheless make an anti-veridical reading more plausible in some cases.)

Because few items (especially in sentence-initial configuration) yielded veridicality ratings in the ambiguous range (around 3), we can assume that higher ratings correspond to more unambiguously veridical items and lower ratings correspond to more ambiguous items. (Unambiguously anti-veridical items would have to have had low ratings.) Our research question then was whether more ambiguous items would show greater negativity in the *before–after* comparison, which would be predicted if the sustained negativity is due to processing difficulty related to the ambiguity introduced by *before*. To test this, we computed item-wise ERPs for each condition (averaging across subjects within each item, rather than vice versa), and subtracted from each *before* ERP the corresponding *after* ERP for that sentence position, yielding difference waves. Then, for each channel and each timepoint, we regressed the amplitude of the sentence-initial *before–after* difference wave on the veridicality ratings, and likewise regressed the amplitude of the sentence-final *before–after* difference wave on the ratings. (We also performed a separate analysis in which we included a quadratic term for the veridicality ratings, in order to account for potential U-shaped effects—e.g., if effects were not monotonically increasing or decreasing, but were negative below the ambiguous '3' region and positive above it, as would be expected if the negativity was largest for ambiguous items but small for both strongly veridical and strongly non-veridical items. This analysis, however, did not yield a significantly better model fit for either sentence position, and thus the quadratic term was removed.)

**Fig 4:**
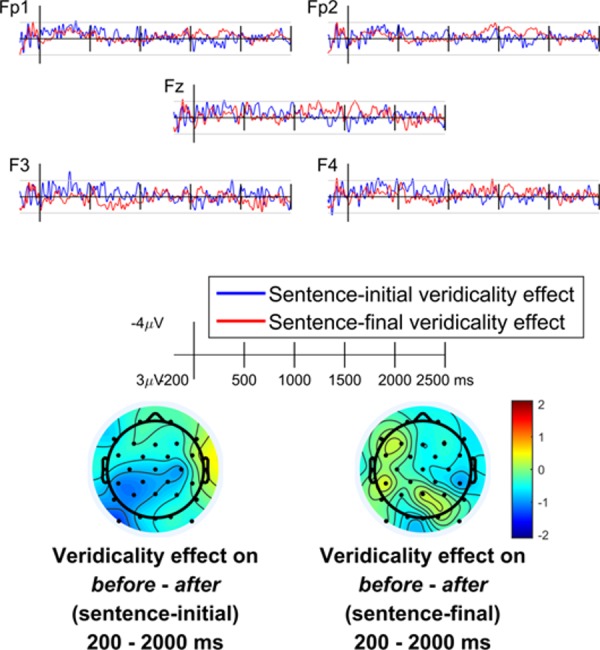
Event-related regression coefficients for the effect of veridicality. *t*-values for the coefficient of veridicality on the amplitude of the *before–after* difference wave in sentence-initial and sentence-final configurations. Horizontal gray lines indicate ±2, the approximate significance thresholds.

The *t* values of the regression coefficients are plotted in Fig 4. It is apparent that there was not a strong trend towards correlation. Early in the time window the sentence-initial difference wave does show a trend towards a negative correlation (see the waveform for F4 and the left posterior portion of the corresponding topographic plot), but this effect is not in the predicted direction: as it is a negative effect, this would mean that the *before–after* difference wave becomes more negative (i.e., there is a larger sustained negativity) as items become more strongly veridical. Furthermore, this effect does not have the same topography as the anterior negativity observed in the main ERP analysis.

## Discussion

This study tested two competing hypotheses about why sentence-initial *before* clauses are more difficult to process than sentence-initial *after* clauses (in, e.g., "Before/after the scientist submitted the article, the journal changed its criteria"), as indexed by an enhanced sustained negative ERP over frontal scalp locations. The traditional account for this effect is that the *before* clauses cause the events in the sentence to be mentioned in a different order than the order they actually occurred in [7]. We compared this to an alternative hypothesis which attributes the difficulty observed in *before* clauses to interpretational ambiguity with respect to whether the event described by *before* actually happened [21, 22]. While these two accounts make the same predictions for temporal clauses in sentence-initial position, they make distinct predictions for clauses in sentence-final position (e.g., "The journal changed its criteria before/after the scientist submitted the article"): the order-of-mention account predicts the effect to reverse, with *after* clauses becoming more difficult than *before* clauses, whereas the account based on interpretational ambiguity does not. In the present experiment, the first to use the ERP method to investigate the processing of temporal connectives in both sentence-initial and sentence-final position, we indeed observed a reversal of the ERP effect: in sentence-initial position, *before* clauses elicited more negative ERPs than *after*, replicating previous findings, whereas in sentence-final position it was *after* that elicited more negative ERPs than *before*. This finding provides support for the traditional order-of-mention account, and suggests that the comprehension of temporal expressions triggers increased processing cost when the mapping between order of linguistic mention and the actual order of events is non-isomorphic.

Although our results provide new evidence to support the order of mention account, they do not rule out the possibility that ambiguity also influences the online comprehension of temporal clauses. While neither the main analysis nor the additional item-wise regression analysis provided evidence to support the hypothesis that the sustained negativity on sentence-initial *before* clauses was due to ambiguity, the study was not originally designed to test a positive prediction of this account. It is possible that the lack of a gradient veridicality effect in the regression analysis occurred because the present study did not contain sufficient variability or sufficient ambiguity to show a veridicality effect, or that explicit metalinguistic ratings were not a sufficiently sensitive indicator of true veridicality bias in these items. Thus, the present study is less strong a test of the veridicality-based account than [21], who directly manipulated veridicality bias. The current result is in fact compatible with an account that allows both the order of mention and ambiguity of veridicality to affect online comprehension. In particular, since ambiguity may have been reduced or absent in some sentence-final temporal clauses, the effect of order may have been more salient in the present study.

On the surface, the manipulation in the present experiment looks somewhat similar to that of a recent fMRI experiment [36] and visual world experiment [36], both of which involved the comprehension of objects in sentences including either *And then…* or *But first…* (e.g., *The squirrel will crack the acorn*. *{And then/But first}*, *it will lick the acorn*.) However, these experiments did not directly examine effects of event order; rather, they examined how objects are represented in different states concurrently. [36] found differences between the neural processing of objects of causative verbs, which are changed as a result of the action described by the verb (e.g., *crack the acorn*), and that of objects of non-causative verbs (e.g., *sniff the acorn*), which is a different question than that examined in the present study (our stimuli included a mixture of causative and non-causative events, and these were not analyzed separately, nor were they intended to be); furthermore, they did not find reliable differences between *and then* and *but first* sentences, which in any case were not the focus of investigation. [37] examined objects that change location as the result of some event (e.g. *The boy will pour the sweetcorn from the bowl into the jar… {And then/but first}*, *he will taste the sweetcorn*.) and found that participants maintain mental representations of the object in multiple places at the same time, and use those representations during comprehension. Again, the *and then*/*but first* manipulation was not central to the research question, and did not trigger robust differences. Therefore, we conclude that the ERP effect observed in the present study is likely reflecting a qualitatively different process than the effects observed in those investigations.

### Working memory

It is surprising that the ERP effects did not reliably correlate with individual differences in participants' working memory. If the sustained negativity for temporal clauses with counter-chronological order of mention is due to the increased load they place on working memory, one might expect the ERP effect to be correlated with a working memory measure. We note, however, that while [21] did indeed replicate the original working memory correlation from [7], [17] did not; therefore, the present study is not the first to find no effect of working memory. It is also worth noting that the working memory cost observed in [21] was hypothesized to be a direct consequence of maintaining veridicality -related ambiguity in memory. To the extent that the current dataset did not find significant evidence to suggest such ambiguity, it may not be surprising that there was also no significant correlation with working memory. It is also possible that, even if sentence-initial *before* and sentence-final *after* clauses are difficult because of counter-chronological order of mention, the specific operations triggered by these clauses are not necessarily based on working memory but perhaps on other operations; for example, realizing an event model which is non-isomorphic with the linguistic input may require actively inhibiting an easier-to-process isomorphic event model (see [38], for an example of a revision-related sustained negativity that failed to significantly correlate with a working memory measure). For this reason, it would be valuable for future research to examine correlations between these ERP effects and other cognitive abilities, such as executive function. We also note that, even if the sustained negativity is based on working memory processes, the nature of the correlation predicted is unclear; while [7] and [21] found negative correlations, such that the effect (a negativity) was largest for participants with high working memory capacity, it seems that a working memory account could just as easily predict positive correlations, under the assumption that processing the challenging counter-chronological order of mention is easier (and thus triggers less sustained negativity) for participants with high working memory than those with low working memory. Given this possibility, we are hesitant to draw strong conclusions about the nature of the sustained negativity based on correlations, or lack thereof, with individual differences.

## Conclusion

In summary, the present study showed direct ERP evidence that conflict between the order in which events are mentioned in a linguistic expression and the order in which the events actually occurred in the world contributes to the processing costs that are observed in the comprehension of temporal clauses. Open questions remain regarding the nature of the cognitive functions underlying this difficulty (e.g., whether temporal clauses that occur out of order trigger working memory operations or other kinds of operations) and the role played by the different semantics of *before* and *after* (especially the ambiguous veridicality of events in a *before* clause). Nonetheless, the current results demonstrate that language comprehenders construct conceptual models of events online as a sentence is unfolding, and that the order of mention of events in a linguistic expression can help or hinder the mapping between a linguistic model and a conceptual model of the world.

## Supporting information

S1 Text Time-window statistics. (DOCX)

S2 Text Re-analysis of EEG data cleaned using ICA. (DOCX)

S1 Spreadsheet Participant information. Demographic information, numbers of trials left per condition for each participant in the ERP analysis, and working memory recall and accuracy scores.
(XLSX)

S2 Spreadsheet Critical stimuli. Highlighted rows indicate items that were later removed from the analysis. Veridicality bias ratings for each item are also given; for a description of how these were collected see the section "The role of veridicality".
(XLSX)
