# Supplementary material for: "Before" and "after": Investigating the relationship between temporal connectives and chronological ordering using event-related potentials
Source: PLoS One. 2017 Apr 3;12(4):e0175199. doi: 10.1371/journal.pone.0175199 (PMC5378364; doi:10.1371/journal.pone.0175199)
Supplement: S1 Text — (DOCX) [file pone.0175199.s001.docx]

For ease of comparison with previous studies, we conducted a traditional statistical analysis on MMN mean amplitudes over the 200-2000 ms time window, grouped into several regions of interest, were compared using repeated measures analyses of variance (ANOVAs) with the factors Connective (2 levels: *before*, *after*), Structure (2 levels: sentence-initial temporal clause, sentence-final temporal clause), and Region (8 levels: frontal [Fp1, Fp2], left anterior [F7, F3, FC5], mid anterior [FC1, FC2, Fz], right anterior [F8, F4, FC6], left posterior [CP5, P7, P3], mid posterior [CP1, CP2, Pz], right posterior [CP6, P8, P4], and occipital [O1, O2, Oz]). Degrees of freedom for *F*-tests involving the factor Region were subjected to the Greenhouse-Geisser correction.

The ANOVA over the mean amplitudes from 200-2000 ms revealed a marginal Connective×Structure×Region interaction (*F*(7,133) = 3.09, *p* = .062).^^[[1]](#footnote-1)^^ We resolved this interaction by examining the Connective×Structure interaction within each region. In the left anterior region there was a significant Connective×Structure interaction (*F*(1,19) = 4.80, *p* = .041); in the other regions there were either no significant effects, or only main effects of Structure, which are not of interest (see footnote 1).Within the left anterior region, the simple effect of Connective was non-significant for sentence-initial clauses (*F*(1,19) = 1.01, *p* = .327) but was significant for sentence-final clauses (*F*(1,19) = 4.96, *p* = .038). While the effect of Connective surprisingly did not reach significance for sentence-initial temporal clauses (contra previous studies, which this effect is intended to replicate), perhaps because of the small number of participants and items (Xiang et al., 2014, included 28 participants and 40 items per condition; Münte et al., 1998, 24 participants and 60 items; Nieuwland, in press, 60 and 30) and perhaps because of the way we divided the electrode array into regions, it was numerically negative as expected (*t*(19) = -1.01), whereas the significant effect of Connective for sentence-final temporal clauses was positive (*t*(19) = 2.23).

1. There was also a significant Structure×Region interaction (*F*(7,133) = 12.81, *p* > .001), which is not of interest because it involves direct comparison across clauses at different portions of the sentence (which differ on a number of factors that were not of interest, such as how far linearly they are into the sentence), and a main effect of Region (*F*(7,133) = 4.83, *p* = .024), which is not of interest because it merely represents differences in the amplitude of the ERP at different portions of the scalp regardless of linguistic manipulations. [↑](#footnote-ref-1)
