# Supplementary material for: "Before" and "after": Investigating the relationship between temporal connectives and chronological ordering using event-related potentials
Source: PLoS One. 2017 Apr 3;12(4):e0175199. doi: 10.1371/journal.pone.0175199 (PMC5378364; doi:10.1371/journal.pone.0175199)
Supplement: S2 Text — (DOCX) [file pone.0175199.s002.docx]

**Working memory analysis**

Following previous ERP studies on temporal connective processing, we also test whether the observed ERP effects correlate with individual differences in working memory.

*Procedure.* After the end of the EEG experimental session and after being given an opportunity to wash their hair, participants completed a computer-mediated version of the reading span task described by Kane, Hambrick, Tuholski, Wilhelm, Payne, and Engle (2004; see also Conway, Kane, Bunting, Hambrick, Wilhelm, & Engle, 2005) to measure individual differences in working memory. The task was administered using Paradigm (Perception Research Systems, Inc.). Participants saw 12 item sets, each consisting of two to five trials. On each trial, the participant saw a visually-presented sentence followed by a "?" and a capital letter. Sentences were either conceptually anomalous (e.g., "During the week of final spaghetti, I felt like I was losing my mind") or conceptually acceptable (e.g., "During the winter you can get a room at the beach for a very low rate"). The participants' task was to read the sentence aloud and then make an acceptability judgment using the mouse. After making the judgment, the participant was to say the following letter aloud, after which the next trial was presented. After completing all two to five trials in an item set, the participant was asked to recall the final letters of each trial in that item set, in order. Item sets and trials were presented in the same order for all participants. Within an item set, no two trials had the same letter following the sentence. Before beginning the test, participants completed a practice block consisting of three two-trial item sets.

*Data analysis.* Each participant's performance on the recall portion of the span task was scored according to the partial-credit unit scoring procedure described by Conway and colleagues (2005). In this procedure, each item set gets a score reflecting what proportion of trials the participant recalled correctly in that item set (e.g., a participant correctly recalling 2 trials out of 5 would receive a score of .4 for that item set) and the scores of the 15 items are then averaged, yielding an aggregate score between 0 and 1 for each participant, with higher scores reflecting greater recall accuracy. Each participant's accuracy on the secondary processing task (acceptability judgments) was also calculated as the proportion of trials with correct performance. Data for one participant who achieved perfect accuracy but was placing his fingers on the keyboard to remember the letters, was replaced with the mean of other participants' scores. Finally, recall and processing scores were averaged to yield a composite score, which was then sphered. Individual participants' recall and accuracy scores are shown in Supplementary File 1.

*Results*. The relationship between individual working memory and the Connective effect (operationalized as the mean amplitude of the before – after difference within the marginal interaction cluster, calculated separately for sentence-initial and sentence-final temporal clauses) is illustrated in Figure 1 of the main text. While there was an apparent numerical trend towards opposite memory effects for sentence-initial versus sentence-final clauses, none of these were significant. Specifically, there was no significant correlation between working memory and effect size either for sentence-initial temporal clauses (b = 0.28, R2 = .02, F(1,18) = 0.46, p = .508) or for sentence-final temporal clauses (b = 0.25, R2 = .03, F(1,18) = 0.57, p = .461), nor did Structure and working memory interact in a linear mixed model with random intercepts for participants (χ2(1) = 1.76, p = .185). Furthermore, the trends were in the opposite direction of those reported earlier: here, participants with higher working memory had effects nearer to zero, whereas for Münte et al. (1998) and Xiang et al (2014) participants with higher working memory had larger (more negative) effects.

*Discussion*. It is surprising that the ERP effects did not reliably correlate with individual differences in participants' working memory. If the sustained negativity for temporal clauses with non-isomorphic order-of-mention is due to the increased load they place on working memory, one might expect the ERP effect to be correlated with a working memory measure. We note, however, that while Xiang and colleagues (2014) did indeed replicate the original working memory correlation from Münte et al. (1998), Nieuwland (2015) did not; therefore, the present study is not the first to find no effect of working memory. It is possible that, even if sentence-initial before and sentence-final after clauses are difficult because of non-isomorphic order-of-mention, the specific operations triggered by these clauses are not necessarily based on working memory but perhaps on other operations; for example, realizing an event model which is non-isomorphic with the linguistic input may require actively inhibiting an easier-to-process isomorphic event model (see Pijnacker et al., 2011, for an example of a revision-related sustained negativity that failed to correlate with a working memory measure). For this reason, it would be valuable for future research to examine correlations between these ERP effects and other cognitive abilities, such as executive function. We also note that, even if the sustained negativity is based on working memory processes, the nature of the correlation predicted is unclear; while Münte et al. (1998) and Xiang et al. (2014) found negative correlations, such that the effect (a negativity) was largest for participants with high working memory capacity, it seems that a working memory account could just as easily predict positive correlations, under the assumption that processing the non-isomorphic order of mention is easier (and thus triggers less sustained negativity) for participants with high working memory than those with low working memory. Given this possibility, we are hesitant to draw strong conclusions about the nature of the sustained negativity based on correlations, or lack thereof, with individual differences.

**Veridicality analysis**

While the main ERP analysis did not show evidence that the sustained negativity on sentence-initial *before* clauses was due to ambiguity in the veridicality of the clause, we hypothesized that such an ambiguity effect may be observable on an item-to-item level if we took into account variation in the veridicality bias of each item. To that end, we collected norming data to evaluate each item's likelihood of being interpreted as veridical, and regressed the item-wise ERP averages on these ratings to see whether more ambiguous items would yield more negative ERP effects.

*Procedure and analysis*. Ratings were collected via Amazon Mechanical Turk, with each item being presented as a single Human Intelligence Task (HIT). In each HIT, participants were shown the sentence up to the end of the temporal clause (which means they were shown a sentence fragment in the case of sentence-initial temporal clauses, but shown a full sentence in the case of sentence-final temporal clauses) and asked to evaluate, on a scale from 1 (very unlikely) to 5 (very likely), the likelihood that the event described in the temporal clause indeed happened. Each HIT was completed by six unique workers, and a worker was allowed to complete multiple HITs, such that one worker may have contributed responses to multiple items, but may only have contributed one response to any given item. Items were normed in both their sentence-initial *before* versions and their sentence-final *before* versions, but not in either *after* version, since we did not predict variation in veridicality bias for *after* sentences. Overall, 1920 ratings were collected (160 items × 2 conditions × 6 workers), 1412 of which were from monolingual English speakers (according to self-report); the others were removed from further analysis. In order to exclude workers from contributing ratings to an item which they had already rated in the other condition, the HITs were divided into two Latin square lists which were presented on two subsequent weekdays, at about the same time of day (08:00-9:00 EST). For each item, the ratings from the self-reported monolingual English speakers were averaged to yield an average veridicality bias rating for that item

*Ratings*. Violin plots of the item-wise average ratings are shown in Figure 1; the ratings for each item are given in Supplementary File 2. It is apparent that sentence-final temporal clauses elicited slightly higher veridicality ratings on average than sentence-initial ones (4.25 vs. 4.19), although this difference was not significant (*t*(159) = 0.94, 95% CI = -0.06…0.17, *p* = .347). Sentence-final temporal clauses also had a larger standard deviation of ratings (0.63 vs. 0.49). The wider range of ratings for sentence-final clauses is likely due to the availability of more context (the matrix clause as well as the temporal clause) which, rather than always disambiguating to a veridical reading, may sometimes have biased participants to make non-veridical readings. (Note that, while none of our items explicitly disambiguated to non-veridical readings—i.e., there were no items like "The police defused the bomb before it exploded"—other aspects of the full sentence may nonetheless make a non-veridical reading more plausible in some cases, whereas on the other hand there is no straightforward way for a matrix clause to absolutely disambiguate to the veridical reading.)

**Figure 1.** Violin plots of average veridicality ratings for temporal clauses in sentence-initial and sentence-final positions. 1 indicates a response that the temporal clause event is "very unlikely" to have occurred, and 5 that the event was "very likely".


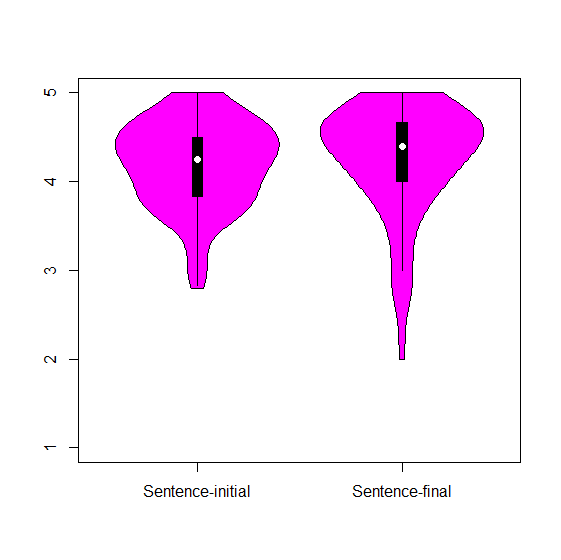


*Regression with ERPs*. Because few items (especially in sentence-initial configuration) yielded veridicality ratings in the ambiguous range (around 3), we can assume that higher ratings correspond to more unambiguously veridical items and lower ratings correspond to more ambiguous items. (Unambiguously non-veridical items would have to have had ratings lower than 3.) Our research question then was whether more ambiguous items would show greater negativity in the *before – after* comparison, which would be predicted if the sustained negativity is due to processing difficulty related to the ambiguity introduced by *before*. To test this, we computed item-wise ERPs for each condition (averaging across subjects within each item, rather than vice versa), and subtracted from each *before* ERP the corresponding *after* ERP for that sentence position, yielding difference waves. Then, for each channel and each timepoint, we regressed the amplitude of the sentence-initial *before – after* difference wave on the veridicality ratings, and likewise regressed the amplitude of the sentence-final *before – after* difference wave on the ratings. (We also performed a separate analysis in which we included a quadratic term for the veridicality ratings, in order to account for potential U-shaped effects—e.g., if effects were not monotonically increasing or decreasing, but were negative below the ambiguous '3' region and positive above it, as would be expected if the negativity was largest for ambiguous items but small for both strongly veridical and strongly non-veridical items. This analysis, however, did not yield a significantly better model fit for either sentence position, and thus the quadratic term was removed.)


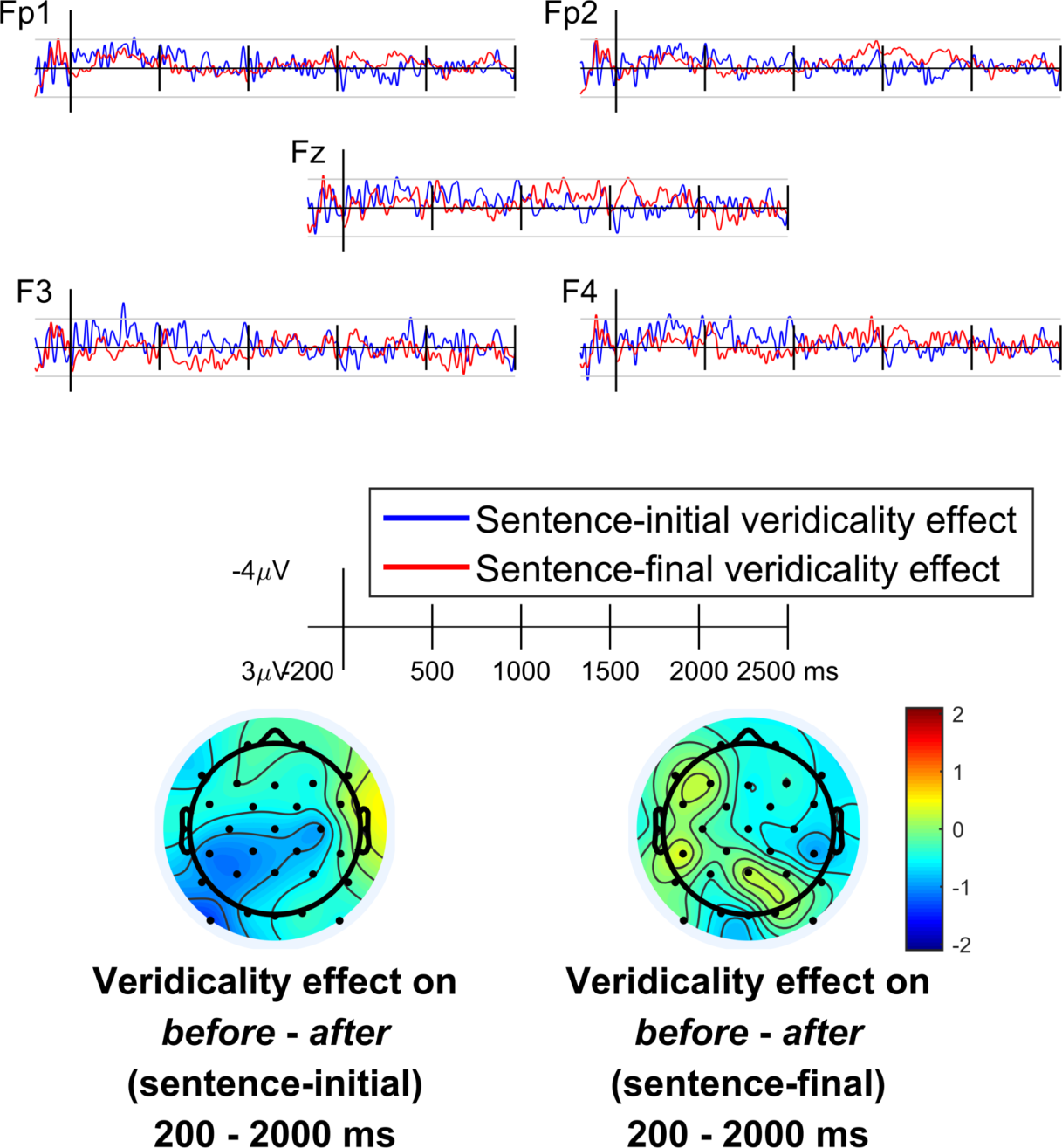


**Figure 2.** *t*-values for the coefficient of veridicality on the amplitude of the *before – after* difference wave in sentence-initial and sentence-final configurations.

*Results*. The *t* values of the regression coefficients are plotted in Figure 2 (horizontal gray lines indicate ±2, the approximate significance thresholds). It is apparent that there was not a strong trend towards correlation. Early in the time window the sentence-initial difference wave does show a trend towards a negative correlation (see the waveform for F4 and the left posterior portion of the corresponding topographic plot), but this effect is not in the predicted direction: as it is a negative effect, this means that the *before – after* difference wave becomes more negative (i.e., there is a larger sustained negativity) as items become more strongly veridical; furthermore, this effect does not have the same topography as the anterior negativity observed in the main ERP analysis.

*Discussion*. Overall, this analysis did not provide evidence to support the hypothesis that the sustained negativity on sentence-initial *before* clauses was due to ambiguity. It is possible that the present stimulus set, in which we did not explicitly manipulate veridicality bias like Xiang et al. (2014) did, did not contain sufficient variability or sufficient ambiguity to show a veridicality effect, or that explicit metalinguistic ratings were not a sufficiently sensitive indicator of true veridicality bias in these items.

**References**

Conway, A., Kane, M., Bunting, M., Hambrick, D., Wilhelm, O., & Engle, R. (2005). Working memory span tasks: a methodological review and user's guide. Psychonomic Bulletin and Review, 12, 769-786.

Kane, M., Hambrick, D., Tuholski, S., Wilhelm, O., Payne, T., & Engle, R. (2004). The generality of working memory capacity: a latent-variable approach to verbal and visuo-spatial memory span and reasoning. *Journal of Experimental Psychology: General, 133*, 189-217.

Pijnacker, J., Geurts, B., van Lambalgen, M., Buitelaar, J., & Hagoort, P. (2011). Reasoning with exceptions: an event-related brain potentials study. *Journal of Cognitive Neuroscience, 23*, 471-480.
